# Got mutants? How advances in chlamydial genetics have furthered the study of effector proteins

**DOI:** 10.1093/femspd/ftaa078

**Published:** 2021-01-29

**Authors:** Shelby E Andersen, Lanci M Bulman, Brianna Steiert, Robert Faris, Mary M Weber

**Affiliations:** Department of Microbiology and Immunology, University of Iowa Carver College of Medicine, Iowa City, IA 52242, USA; Department of Microbiology and Immunology, University of Iowa Carver College of Medicine, Iowa City, IA 52242, USA; Department of Microbiology and Immunology, University of Iowa Carver College of Medicine, Iowa City, IA 52242, USA; Department of Microbiology and Immunology, University of Iowa Carver College of Medicine, Iowa City, IA 52242, USA; Department of Microbiology and Immunology, University of Iowa Carver College of Medicine, Iowa City, IA 52242, USA

**Keywords:** *Chlamydia*, type III secretion, effector, inclusion, inclusion membrane protein, genetics

## Abstract

*Chlamydia trachomatis* is the leading cause of infectious blindness and a sexually transmitted infection. All chlamydiae are obligate intracellular bacteria that replicate within a membrane-bound vacuole termed the inclusion. From the confines of the inclusion, the bacteria must interact with many host organelles to acquire key nutrients necessary for replication, all while promoting host cell viability and subverting host defense mechanisms. To achieve these feats, *C. trachomatis* delivers an arsenal of virulence factors into the eukaryotic cell via a type 3 secretion system (T3SS) that facilitates invasion, manipulation of host vesicular trafficking, subversion of host defense mechanisms and promotes bacteria egress at the conclusion of the developmental cycle. A subset of these proteins intercalate into the inclusion and are thus referred to as inclusion membrane proteins. Whereas others, referred to as conventional T3SS effectors, are released into the host cell where they localize to various eukaryotic organelles or remain in the cytosol. Here, we discuss the functions of T3SS effector proteins with a focus on how advances in chlamydial genetics have facilitated the identification and molecular characterization of these important factors.

## Teaser

In this article, the authors review the function of *Chlamydia trachomatis* secreted effector proteins and Incs, with special emphasis on how the advancement of chlamydial genetics has enabled functional characterization of these important proteins.

## INTRODUCTION

The family *Chlamydiaceae* consists of eleven species of obligate intracellular pathogens that are of human and veterinary importance (Bachmann, Polkinghorne and Timms 2014). *Chlamydia trachomatis* is of particular significance to human health and consists of 15 serovars that can cause multiple disease states of differing severity and associated comorbidities (Elwell, Mirrashidi and Engel 2016). Infection of the conjunctival epithelium with serovars A–C can cause blinding trachoma, the leading cause of non-congenital blindness in the world (Hu, Holland and Burton 2013), while serovars D–K infect a variety of cells belonging to the stratified squamous epithelium of the genital tract and cause the most common sexually transmitted infection in the world. Serovars L1–L3 can initially infect mucosal epithelia of the rectum, external sex organs or even the pharynx manifesting as a type of abscess or ulcer. L1–L3 strains are the etiological agent of lymphogranuloma venereum and have a unique ability to survive within mononuclear phagocytes where they can be trafficked to the draining lymph nodes and proliferate causing disease characterized by lymphadenitis, lymphangitis and formation of buboes (Lausen *et al*. 2018). Due to the large spectrum of diseases caused by the many serovars of *C. trachomatis*, and their substantial impact on human health, understanding the mechanisms of pathogenesis is important for improving global health outcomes.

**Figure 1.**
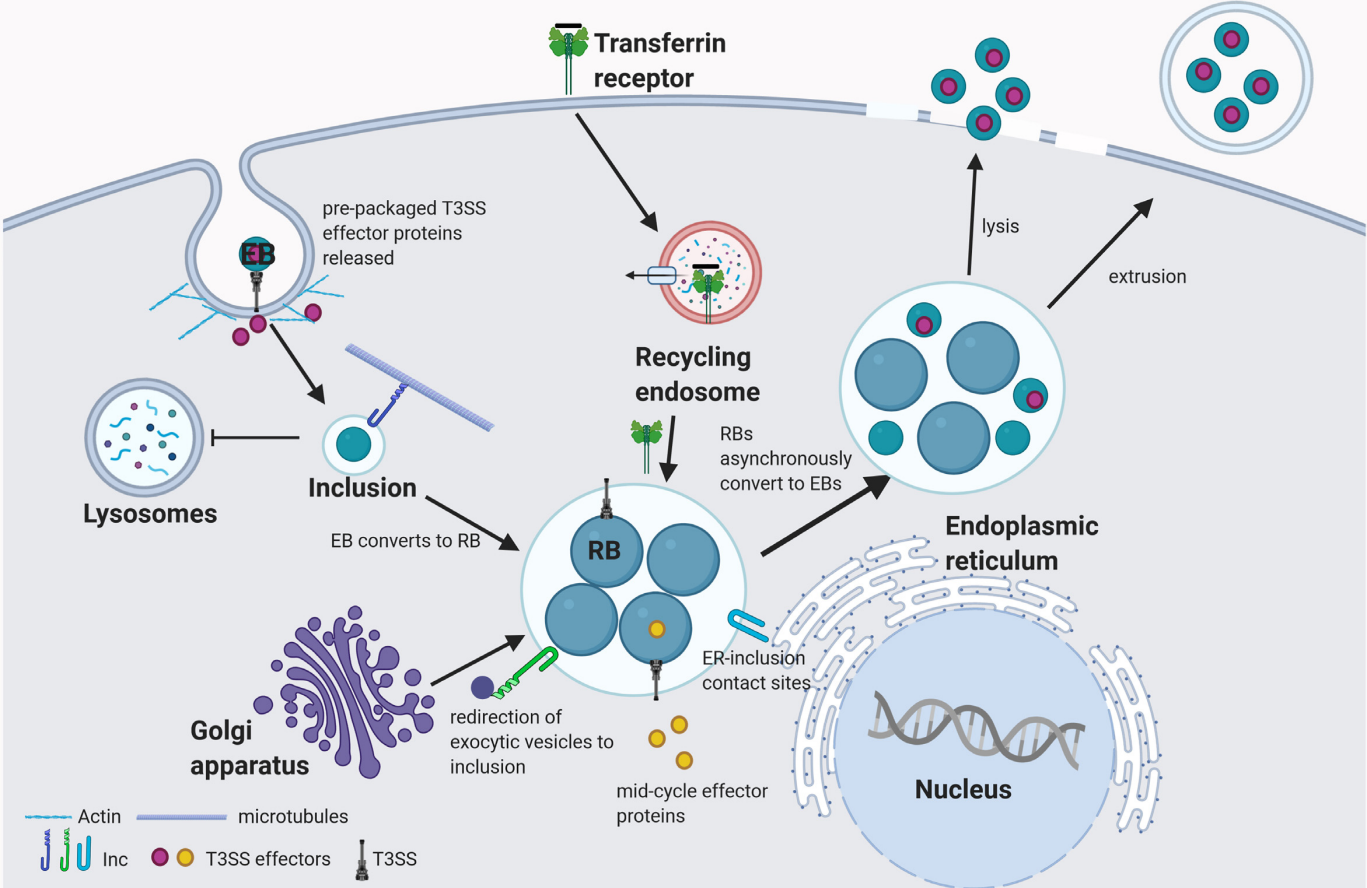
The intracellular life cycle of *Chlamydia*. Contact between the EB and the host cell triggers delivery of pre-packaged effector proteins that trigger cytoskeletal and membrane remodeling events to promote invasion. The nascent inclusion avoids fusion with lysosomes and traffics along microtubules to the MTOC. EBs convert to RBs and replication ensues via polarized cell division. Throughout the infection cycle, additional effector proteins are delivered into the host cell or the inclusion membrane to mediate interactions with various host organelles. RBs undergo asynchronous conversion to EBs and at the conclusion of the developmental cycle, EBs are released by extrusion or lysis.

All chlamydiae share a biphasic developmental cycle in which the bacteria alternate between two distinct forms: the infectious, non-replicative elementary body (EB) and the non-infectious, replicative reticulate body (RB) (Abdelrahman and Belland 2005). Following contact with the host cell, pre-packaged type 3 secretion system (T3SS) effector proteins are delivered into the eukaryotic cell (Saka *et al*. 2011), triggering endocytosis of the EB (Fig. 1). The EB is internalized into a membrane-bound compartment, termed the inclusion, that avoids fusion with lysosomes and instead traffics along microtubules using dynein to the peri-Golgi region (Grieshaber, Grieshaber and Hackstadt 2003; Scidmore, Fischer and Hackstadt 2003). Here, the EBs differentiate into RBs and replicate by polarized cell division (Abdelrahman *et al*. 2016). From the confines of the inclusion, the bacteria will interact with many host organelles to acquire key nutrients necessary for replication all while promoting host cell viability (Scidmore, Fischer and Hackstadt 1996; Derré *et al*. 2007; Pokorzynski, Thompson and Carabeo 2017; Stanhope *et al*. 2017). Following multiple rounds of replication, RBs will undergo asynchronous conversion to EBs, which are released via extrusion or host cell lysis (Hybiske and Stephens 2007).

Throughout the infection cycle, *C. trachomatis* is predicted to deliver over 100 effector proteins into the host cell via its T3SS (Table 1) (Bugalhão and Mota 2019). Among these are the inclusion membrane (Inc) proteins and conventional T3SS (cT3SS) effector proteins. Inc proteins possess a bi-lobed hydrophobic domain of ∼40 amino acids (Bannantine *et al*. 2000), which allows for incorporation into the inclusion membrane in such a way that their N- and C-termini are exposed to the eukaryotic host cell cytosol (Scidmore-Carlson *et al*. 1999). While 58 Incs have been predicted based on the presence of a bi-lobed hydrophobic domain, only 38 have been verified to localize to the inclusion membrane (Table 1) (Weber *et al*. 2015). Conversely, cT3SS effectors are secreted into the host cell where they have been detected at the plasma membrane, nucleus or within the cytosol and are predicted to play critical roles in host cell invasion, nutrient acquisition and immune evasion (Elwell, Mirrashidi and Engel 2016; Bugalhão and Mota 2019). Importantly, a subset of these cT3SS effectors are preloaded into the type III apparatus and presumably play important roles during cellular invasion and early stages of infection. Bioinformatic analysis looking for *C. trachomatis* proteins that possess eukaryotic-like domains or a T3SS signal has identified a large list of candidate effector proteins (Subtil *et al*. 2005; Muschiol *et al*. 2011; Da Cunha *et al*. 2014) (Table 1), many of which are strong secretion candidates based on studies in surrogate hosts. Undoubtedly, both classes of effector proteins, Incs and cT3SSs, are important for *C. trachomatis* pathogenesis, and understanding their form and function gives insight into chlamydial disease.

**Table 1.** *Chlamydia trachomatis* Incs and cT3SS effector proteins and their functions.

D/UW-3CX	L2/434/Bu	Gene name	Identification method/localization	Mutant/phenotype	Host cell target	Function	Reference
CT005	CTL0260	*incV*	IFA/inclusion membrane	TargeTron/reduced VAP recruitment	VAPA/B	Formation of ER-inclusion MCS	(Shaw *et al*. 2000; Weber *et al*. 2015; Stanhope *et al*. 2017; Wang, Hybiske and Stephens 2018)
CT006	CTL0261		IFA/inclusion membrane	None	Unknown	Unknown	(Kokes *et al*. 2015; Weber *et al*. 2015)
CT042	CTL0298	*glgX*	Shigella T3S assay/inclusion lumen	Transposon/unknown	Unknown	glycogen hydrolase	(Gehre *et al*. 2016)
CT053	CTL0309		Yersinia T3S assay/unknown	None	Unknown	Unknown	(Da Cunha *et al*. 2014)
CT082	CTL0338		Yersinia T3S assay/unknown	None	Unknown	Unknown	(Pais *et al*. 2013)
CT083	CTL0338A		Shigella T3S assay/unknown	None	Unknown	Unknown	(Subtil *et al*. 2005)
CT105	CTL0360	*cteG*	IFA/plasma membrane and Golgi	TargeTron/smaller inclusions	Unknown	Unknown	(Pais *et al*. 2019)
CT101	CTL0356	*mrcA*	IFA/inclusion membrane	TargeTron/reduced extrusion	ITPR3	Promotes *Chlamydia* extrusion	(Shaw *et al*. 2000; Mital *et al*. 2010; Nguyen, Lutter and Hackstadt 2018)
CT115	CTL0370	*incD*	IFA/inclusion membrane	None	CERT	Formation of ER-inclusion MCS, non-vesicular lipid acquisition	(Scidmore-Carlson *et al*. 1999; Li *et al*. 2008; Derré, Swiss and Agaisse 2011; Agaisse and Derré 2014; Kokes *et al*. 2015; Weber *et al*. 2015; Kumagai *et al*. 2019)
CT116	CTL0371	*incE*	IFA/inclusion membrane	None	SNX5/6	Manipulates retromer-mediated transport	(Scidmore-Carlson *et al*. 1999; Li *et al*. 2008; Mirrashidi *et al*. 2015; Weber *et al*. 2015; Elwell *et al*. 2017; Paul *et al*. 2017; Sun *et al*. 2017)
CT117	CTL0372	*incF*	IFA/inclusion membrane	None	Unknown	Unknown	(Scidmore-Carlson *et al*. 1999; Li *et al*. 2008; Weber *et al*. 2015)
CT118	CTL0373	*incG*	IFA/inclusion membrane	None	14-3-3β	Unknown	(Scidmore-Carlson *et al*. 1999; Scidmore and Hackstadt 2001; Li *et al*. 2008)
CT119	CTL0374	*incA*	IFA/inclusion membrane	TargeTron/defects in homotypic inclusion fusion	VAMP3/7/8	Homotypic inclusion fusion, regulation of host vesicular trafficking	(Scidmore-Carlson *et al*. 1999; Bannantine *et al*. 2000; Suchland *et al*. 2000; Delevoye *et al*. 2008; Li *et al*. 2008; Paumet *et al*. 2009; Johnson and Fisher 2013; Ronzone and Paumet 2013; Ronzone *et al*. 2014; Weber *et al*. 2016; Wang, Hybiske and Stephens 2018; Cingolani *et al*. 2019)
CT134	CTL0389		IFA/inclusion membrane	None	Unknown	Unknown	(Weber *et al*. 2015)
CT135	CTL0390		IFA/inclusion membrane	None	Unknown	Unknown	(Weber *et al*. 2015)
CT142	CTL0397		Yersinia T3S assay/inclusion lumen	None	Unknown	Unknown	(Da Cunha *et al*. 2014, 2017)
CT143	CTL0398		Yersinia T3S assay/inclusion lumen	Transposon/unknown	Unknown	Unknown	(Da Cunha *et al*. 2014, 2017; LaBrie *et al*. 2019)
CT144	CTL0399		Yersinia T3S assay/inclusion lumen	None	Unknown	Unknown	(Da Cunha *et al*. 2014, 2017)
CT147	CTL0402		IFA/inclusion membrane	None	Unknown	Unknown	(Belland *et al*. 2003; Li *et al*. 2008; Weber *et al*. 2015)
CT156	absent	*lda1*	IFA/lipid droplets	None	Unknown	Unknown	(Kumar *et al*. 2006)
CT161	CTL0417		Yersinia T3S assay/unknown	None	Unknown	Unknown	(Da Cunha *et al*. 2014)
CT163	CTL0419	*lda2*	IFA/lipid droplets	None	Unknown	Unknown	(Kumar *et al*. 2006)
CT179	CTL0431		IFA/inclusion membrane	TargeTron/none	Unknown	Unknown	(Weber *et al*. 2015, 2017)
CT192	CTL0444		IFA/inclusion membrane	None	Unknown	Unknown	(Weber *et al*. 2015)
CT203	CTL0455		Shigella T3S assay/unknown	None	Unknown	Unknown	(Subtil *et al*. 2005)
CT222	CTL0475		IFA/inclusion membrane	None	Unknown	Unknown	(Bannantine *et al*. 2000; Shaw *et al*. 2000; Li *et al*. 2008; Weber *et al*. 2015)
CT223	CTL0476	*ipaM*	IFA/inclusion membrane	None	Cep170	Hijacks microtubule organizing functions and controls microtubule assembly	(Bannantine *et al*. 2000; Shaw *et al*. 2000; Li *et al*. 2008; Alzhanov *et al*. 2009; Dumoux *et al*. 2015; Weber *et al*. 2015)
CT224	CTL0477		IFA/inclusion membrane	TargeTron/none	Unknown	Unknown	(Shaw *et al*. 2000; Alzhanov *et al*. 2009; Weber *et al*. 2015)
CT225	CTL0477A		IFA/inclusion membrane	None	Unknown	Unknown	(Shaw *et al*. 2000; Li *et al*. 2008; Weber *et al*. 2015)
CT226	CTL0478		IFA/inclusion membrane	None	LRRF1	Unknown	(Shaw *et al*. 2000; Li *et al*. 2008; Weber *et al*. 2015; Olson *et al*. 2019)
CT227	CTL0479		IFA/inclusion membrane	None	Unknown	Unknown	(Shaw *et al*. 2000; Weber *et al*. 2015)
CT228	CTL0480		IFA/inclusion membrane	TargeTron	MYP1	Inhibits chlamydial extrusion	(Shaw *et al*. 2000, 2018; Li *et al*. 2008; Lutter *et al*. 2013)
CT229	CTL0481	*cpoS*	IFA/inclusion membrane	TargeTron, chemical/growth defect and premature inclusion lysis	Rab1/4/6/8/10/14/18/33/34/35	Manipulation of host vesicular trafficking	(Bannantine *et al*. 2000; Shaw *et al*. 2000; Rzomp, Moorhead and Scidmore 2006; Li *et al*. 2008; Weber *et al*. 2015, 2017; Sixt *et al*. 2017; Faris *et al*. 2019)
CT232	CTL0484	*incB*	IFA/inclusion membrane	None	Unknown	Unknown	(Bannantine *et al*. 2000; Li *et al*. 2008; Mital *et al*. 2010; Weber *et al*. 2015)
CT233	CTL0485	*incC*	IFA/inclusion membrane	TargeTron/growth defect and premature inclusion lysis	Unknown	Unknown	(Bannantine *et al*. 2000; Li *et al*. 2008; Weber *et al*. 2015, 2017)
CT249	CTL0500A		IFA/inclusion membrane	None	Unknown	Unknown	(Shaw *et al*. 2000; Jia *et al*. 2007; Li *et al*. 2008)
CT288	CTL0540		IFA/inclusion membrane	TargeTron/growth defect	CCDC146	Unknown	(Bannantine *et al*. 2000; Li *et al*. 2008; Weber *et al*. 2015, 2017; Almeida *et al*. 2018)
CT345	CTL0599		IFA/inclusion membrane	None	Unknown	Unknown	(Weber *et al*. 2015)
CT358	CTL0612		IFA/inclusion membrane	None	Unknown	Unknown	(Li *et al*. 2008)
CT383	CTL0639		IFA/inclusion membrane	TargeTron/growth defect and premature inclusion lysis	Unknown	Unknown	(Weber *et al*. 2015, 2017)
CT429	CTL0688		Yersinia T3S assay/unknown	None	Unknown	Unknown	(Da Cunha *et al*. 2014)
CT440	CTL0699		IFA/inclusion membrane	None	Unknown	Unknown	(Li *et al*. 2008)
CT442	CTL0701	*crpA*	IFA/inclusion membrane	None	Unknown	Unknown	(Bannantine *et al*. 2000; Li *et al*. 2008; Weber *et al*. 2015)
CT449	CTL0709		IFA/inclusion membrane	TargeTron/growth defect	Unknown	Unknown	(Weber *et al*. 2015, 2017)
CT456	CTL0716	*tarP*	*Chlamydia* BlaM assay/T3S dependent/cytosol near inclusion	FRAEM, TargeTron/invasion defect	Vinculin, FAK, Rac GEFs (Sos1, Vav2)	Host cell invasion	(Clifton *et al*. 2004; Jewett *et al*. 2006, 2008; Lane *et al*. 2008; Mehlitz *et al*. 2010; Jiwani *et al*. 2012, 2013; Mueller and Fields 2015; Thwaites *et al*. 2015; Parrett *et al*. 2016; Ghosh *et al*. 2018, 2020; Whitewood *et al*. 2018; Faris *et al*. 2020)
CT473	CTL0734	*lda3*	IFA/lipid droplets	None	Unknown	Unknown	(Kumar *et al*. 2006)
CT483	CTL0744		IFA/inclusion membrane	None	Unknown	Unknown	(Shaw *et al*. 2000)
CT529	CTL0791		IFA/inclusion membrane	None	Unknown	Unknown	(Li *et al*. 2008)
CT550	CTL0812		Shigella T3S assay/unknown	Transposon/unknown	Unknown	Unknown	(Subtil *et al*. 2005; LaBrie *et al*. 2019)
CT565	CTL0828		IFA/inclusion membrane	None	Unknown	Unknown	(Shaw *et al*. 2000)
CT606.1	CTL0870		Shigella T3S assay/unknown	None	Unknown	Unknown	(Subtil *et al*. 2005)
CT610	CTL0874	*cadD*	Shigella T3S assay/unknown	None	Unknown	Unknown	(Subtil *et al*. 2005; Kokes *et al*. 2015)
CT618	CTL0882		IFA/inclusion membrane	None	Unknown	Unknown	(Li *et al*. 2008)
CT619	CTL0883		Shigella T3S assay/unknown	None	Hrs, tsg101	Unknown	(Muschiol *et al*. 2011; Vromman *et al*. 2016)
CT620	CTL0884		Shigella T3S assay/cytosol	None	Hrs	Unknown	(Muschiol *et al*. 2011; Vromman *et al*. 2016)
CT621	CTL0885		Shigella T3S assay/cytosol	None	Hrs	Unknown	(Hobolt-Pedersen *et al*. 2009; Muschiol *et al*. 2011; Vromman *et al*. 2016)
CT622	CTL0886		Shigella T3S assay/cytosol	TargeTron/growth and inclusion defect	Unknown	Unknown	(Gong *et al*. 2017; Cossé *et al*. 2018)
CT652.1	CTL0021		Shigella T3S assay/unknown	None	Unknown	Unknown	(Subtil *et al*. 2005)
CT656	CTL0025		Yersinia T3S assay/unknown	None	Unknown	Unknown	(Da Cunha *et al*. 2014)
CT694	CTL0063	*tmeA*	*Chlamydia* BlaM assay/inclusion and plasma membrane	Transposon, FRAEM/invasion defect	AHNAK, N-WASP	Host cell invasion	(Hower, Wolf and Fields 2009; Bullock, Hower and Fields 2012; Mueller and Fields 2015; McKeun *et al*. 2017; Wang, Hybiske and Stephens 2018; LaBrie *et al*. 2019; Faris *et al*. 2020)
CT695	CTL0064	*tmeB*	*Chlamydia* BlaM assay/inclusion and plasma membrane	FRAEM/no defect	Unknown	Unknown	(Hower, Wolf and Fields 2009; Mueller and Fields 2015)
CT711	CTL0080		Shigella T3S assay/nucleus	None	Hrs	Unknown	(Muschiol *et al*. 2011; Vromman *et al*. 2016)
CT712	CTL0081		Shigella T3S assay/unknown	None	Hrs	Unknown	(Muschiol *et al*. 2011; Vromman *et al*. 2016)
CT718	CTL0087		Shigella T3S assay/unknown	None	Unknown	Unknown	(Subtil *et al*. 2005)
CT737	CTL0106	*nue*	Shigella T3S assay/nucleus	None	H2B, H3 and H4	Histone methyltransferase	(Pennini *et al*. 2010)
CT738	CTL0107		Shigella T3S assay/unknown	None	Unknown	Unknown	(Subtil *et al*. 2005)
CT798	CTL0167	*glgA*	IFA/inclusion lumen and cytosol	None	Unknown	Glycogen synthase	(Lu *et al*. 2013)
CT813	CTL0184	*inaC*	IFA/inclusion membrane	TargeTron, chemical/F-actin recruitment defect	14-3-3 proteins, ARF1/4, VAMP7/8	Modulates post-translational modification of microtubules, controls Golgi complex positioning at the inclusion,	(Shaw *et al*. 2000; Chen *et al*. 2006; Delevoye *et al*. 2008; Li *et al*. 2008; Kokes *et al*. 2015; Wesolowski *et al*. 2017) Wesolowski, Chen C, Kokes, Li, Shaw, Delevoye
CT847	CTL0219		Yersinia T3S assay/unknown	None	GCIP	Unknown	(Chellas-Géry, Linton and Fields 2007)
CT848	CTL0220		Shigella T3S assay/unknown	None	Unknown	Unknown	(Subtil *et al*. 2005)
CT849	CTL0221		Yersinia T3S assay/unknown	None	Unknown	Unknown	(Da Cunha *et al*. 2014)
CT850	CTL0223		IFA/inclusion membrane	TargeTron/none	DYNLT1	Positioning of inclusion at centrosomal region	(Shaw *et al*. 2000; Mital *et al*. 2010, 2015)
CT875	CTL0255	*tepP*	*C.t*. chaperone IP/cytosol near inclusion	Chemical, TargeTron/growth defect	CRK, CRKL, GSK3B, PI3K,	Regulates innate immune signaling early in infection	(Chen *et al*. 2014; Carpenter *et al*. 2017; Dolat and Valdivia 2020)

**Figure 2.**
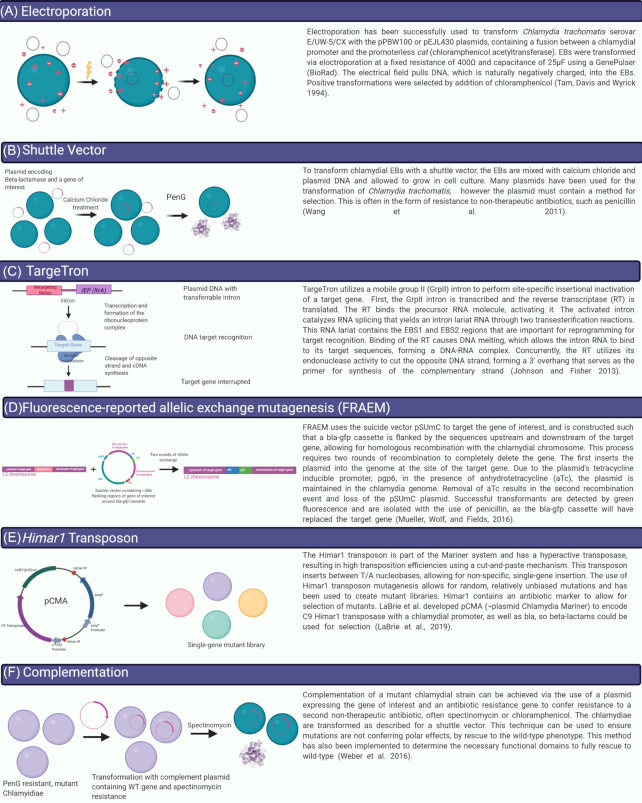
Advances in chlamydial genetics. **(A)** Initial experiments to transform chlamydiae used electroporation; however, **(B)***C. trachomatis* serovar L2 is routinely transformed via chemical transformation with calcium chloride. Using the endogenous L2 plasmid fused to an *E. coli* plasmid, a *C. trachomatis* L2 shuttle vector was developed. This plasmid possesses a GFP fluorescent marker, antibiotic selection marker (*bla*), an *E. coli* origin of replication and a multiple cloning site (MCS). The shuttle vector is routinely used to express epitope-tagged effector proteins in *C. trachomatis* L2. **(C)**The group II intron (TargeTron) approach enables site-specific gene disruption via LtrA. LtrA reverse transcribes and splices the intron into the target site in the recipient's DNA, resulting in insertional inactivation of the target. **(D)** Site-specific mutagenesis via fluorescence-reported allelic exchange mutagenesis (FRAEM) uses the shuttle vector pSU6 to disrupt the target gene of interest. In the absence of tetracycline, pSU6 behaves as a suicide vector. **(E)** The *Himar1* transposase randomly inserts between T/A nucleotides, resulting in non-specific gene inactivation.**(F)** Mutants generated via any of the aforementioned techniques can be complemented using pBomb3, pBomb4 or pSU6.

Historically, chlamydiae have been recalcitrant to genetic manipulation owing to its obligate intracellular lifestyle, biphasic developmental cycle and limited metabolic activity of EBs (Bastidas and Valdivia 2016; Hooppaw and Fisher 2016; Rahnama and Fields 2019). While the first publication reporting transient transformation of *Chlamydia* using electroporation occurred in 1994 (Tam, Davis and Wyrick 1994), it would be another 17 years before *Chlamydia* was stably transformed with a shuttle vector (Fig. 2A and B). In 2011, Wang *et al*. reported an *Escherichia coli–C. trachomatis* shuttle vector that could be stably introduced into *C. trachomatis* serovar L2 using CaCl_2_, and transformants could be selected for using penicillin G (Wang *et al*. 2011). Since that landmark study, the *C. trachomatis* shuttle vector has been modified to include additional fluorescent markers, inducible promoters and various epitope tags, providing a practical tool for the rapid identification of secreted effectors and Incs (Agaisse and Derré 2013; Wickstrum *et al*. 2013; Bauler and Hackstadt 2014; Mueller and Fields 2015; Weber *et al*. 2015). Soon thereafter, the TargeTron (Millipore Sigma St. Louis, MO), a mobile group II intron system, was used to generate site-specific mutations in *C. trachomatis* (Johnson and Fisher 2013) (Fig. 2C) and was also used to create the first site-specific double mutants (Lowden *et al*. 2015). Gene deletion by fluorescence-reported allelic exchange mutagenesis (FRAEM) was developed as an alternative method to generate site-specific *C. trachomatis* mutants (Mueller, Wolf and Fields 2016) (Fig. 2D). Recent adaptions to FRAEM allow for the generation of markerless gene deletions (Keb, Hayman and Fields 2018) that, importantly, can overcome potential polar effects associated with other genetic disruption systems. Additionally, systems for random mutagenesis using the *Himar* transposon have been described and are now being used to generate *C. trachomatis* mutant libraries (LaBrie *et al*. 2019) (Fig. 2E). Complementation of *C. trachomatis* mutants can also be achieved using two vector systems, pSU6 and pBomb3, and has been used to link effector functions to mutant phenotypes (Mueller, Wolf and Fields 2016; Weber *et al*. 2016; Faris *et al*. 2019) (Fig. 2F). The ability to express epitope-tagged proteins in *Chlamydia*, as well as the development of systems for site-specific or random chemical mutagenesis, (Kari *et al*. 2011; Nguyen and Valdivia 2012; Kokes *et al*. 2015), has revolutionized what we know about how *C. trachomatis* manipulates the host cell. In this review, we will discuss the function of select Incs and cT3SS effector proteins with emphasis on how newly developed genetic tools have enabled functional characterization of these important factors.

## HOST CELL INVASION

As an obligate intracellular bacterium, invasion of a host cell is paramount to chlamydial replication and initiation of human disease. Contact between the susceptible host cell and the EB triggers delivery of pre-packaged T3SS effector proteins that induce cytoskeletal rearrangements and plasma membrane remodeling events that promote EB internalization via filopodial capture and macropinocytosis-like pathways (Ford *et al*. 2018).

**Figure 3.**
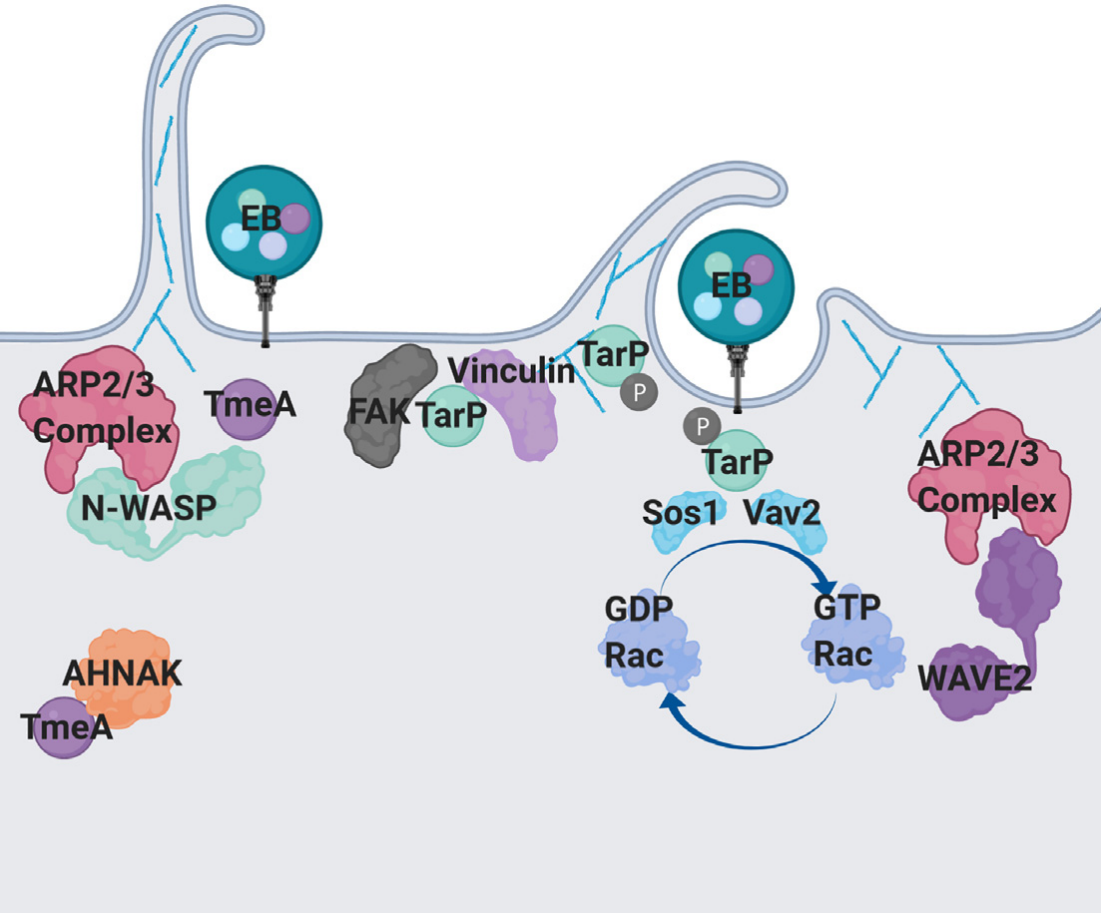
TarP and TmeA, mediators of host cell invasion. TmeA and TarP are pre-packaged in EBs and are delivered into the host cell to promote invasion. TarP is phosphorylated upon entry into the host cell where it binds to Rac GEFs (SOS and VAV2) to activate the small GTPase Rac, which results in ARP2/3-dependent actin branching via complex activation by the NPF WAVE2. TarP can also directly bundle and polymerize actin. Focal adhesion proteins, FAK and vinculin, are also bound by TarP and may serve to promote cell adherence to the extracellular matrix. TmeA binds to the NPF N-WASP to promote ARP2/3-dependent actin branching and filopodia capture of EBs. TmeA also binds AHNAK, which may serve a post-invasion role.

Successful infiltration of a host cell by *C. trachomatis* EBs induces protein tyrosine phosphorylation of numerous host proteins at the EB invasion site (Birkelund, Johnsen and Christiansen 1994; Fawaz *et al*. 1997). Immunoprecipitation of infected host cell lysates using an anti-phosphotyrosine antibody coupled with mass spectrometry identified the presence of a bacterial effector protein, CT456, now designated translocated actin-recruiting phosphoprotein (TarP) (Clifton *et al*. 2004). Depending on the serovar, the N-terminal region of TarP contains 1–12 copies of a tyrosine phosphodomain that is phosphorylated by the eukaryotic kinases p60-src, Yes, Fyn and Abl (Carlson *et al*. 2005; Clifton *et al*. 2005; Elwell *et al*. 2008; Jewett *et al*. 2008; Lutter *et al*. 2010). N-terminal tyrosine phosphorylation of TarP enables interactions with numerous host proteins, including two guanine nucleotide exchange factors (GEFs), Vav2 and Sos1, which mediate GTP exchange on the small GTPase Rac (Lane *et al*. 2008) (Fig. 3). Intriguingly, Rac was shown to be important for host cell invasion through activation of the nucleation promoting factor (NPF) WAVE2 and subsequent recruitment of the ARP2/3 complex (Carabeo *et al*. 2002, 2007; Lane *et al*. 2008).

TarP is also able to recruit F-actin to the invasion site in the absence of tyrosine phosphorylation (Clifton *et al*. 2005) (Fig. 3). Detailed biochemical analysis of TarP revealed it possesses C-terminal filamentous (F)-actin and globular (G)-actin binding sites (Jewett *et al*. 2006, 2010; Jiwani *et al*. 2013; Ghosh *et al*. 2018). These regions allow TarP to bind and bundle actin in the absence of host factors. However, it can cooperate with the ARP2/3 complex to increase the rate of actin polymerization (Jewett *et al*. 2006; Jiwani *et al*. 2012).

In aggregate, these studies suggest that TarP's actin binding domain may promote extension of the initial actin filament while TarP's N-terminal phosphodomains bind Rac regulatory proteins for ARP2/3 complex recruitment required for actin branching. Recent advances in chlamydial genetics allowed for generation of a TarP mutant and subsequent confirmation of its role in host cell invasion (Ghosh *et al*. 2020). Interestingly, complementation of the mutant with domain mutants revealed that the F-actin binding domains are necessary for host cell invasion, whereas lack of tyrosine phosphodomains only minimally impaired invasion (Ghosh *et al*. 2020), suggesting *C. trachomatis* may have alternative methods to induce ARP2/3-mediated actin branching events needed for host cell invasion.

TarP has also been shown to play an important role in recruiting focal adhesion kinase (FAK) to the site of chlamydial invasion by mimicking the leucine–aspartate (LD) motifs (LD*X*LL*XX*L) found in the host protein paxillin (Thwaites *et al*. 2014) (Fig. 3). Using an EPEC-based heterologous system in which TarP or TarP deletion constructs were fused to translocated intimin receptor (Tir), Thwaites *et al*. demonstrated that the LD motif of TarP binds FAK to the same degree as the LD2 motif of paxillin (Thwaites *et al*. 2015). Genetic elimination of FAK or conservative mutation of the leucine residues of TarP's LD motif abolishes actin recruitment and cell signaling.

RNA interference screens have shown that vinculin is necessary for *C. trachomatis* replication (Elwell *et al*. 2008; Gurumurthy *et al*. 2010), and recent molecular studies revealed vinculin is also important for F-actin assembly at the plasma membrane to promote invasion (Thwaites *et al*. 2015). Vinculin contains two domains, Vh1 and Vt, that when bound maintain vinculin in a closed, inactive conformation. Binding of specific proteins, such as talin, relieves vinculin autoinhibition, resulting in activation. Talin activation of vinculin at the site of focal adhesions serves to link actin filaments of the cytoskeleton with membrane-bound extracellular facing integrins to mediate interactions with the extracellular matrix (DeMali, Jue and Burridge 2006). Notably, TarP contains three vinculin binding domains (VBDs) in its C-terminus with VBD1 being necessary for binding and recruitment of vinculin (Thwaites *et al*. 2015). Using the EPEC-Tir system, this motif alone was shown to be sufficient to induce actin recruitment to the plasma membrane in a vinculin-dependent and FAK-independent manner (Fig. 3).

Collectively, these studies indicate that TarP may mediate the formation of pseudo-focal adhesion structures at the invasion site via recruitment of FAK and vinculin, which modulate the cytoskeletal network. Indeed, infection of host cells with multiple chlamydial species and serovars increases the number of focal adhesions, which has recently been linked to TarP and its interaction with vinculin (Pedrosa *et al*. 2020). Presumably, this interaction acts to stabilize focal adhesions, increasing adhesion to the extracellular matrix to maintain *Chlamydia* infection in a high-turnover environment (Pedrosa *et al*. 2020). Importantly, the recent generation of a TarP mutant will now allow researchers to directly connect TarP-vinculin interactions to stabilizing focal adhesions.

Delivery of TarP into host cells does not require *de novo* bacterial protein synthesis (Clifton *et al*. 2004), suggesting proteins produced by RBs could be retained and primed for type III secretion by EBs. This critical observation gave rise to the idea that genes transcribed late in the *C. trachomatis* infection cycle could be effector proteins that promote invasion or early events in the developmental cycle (Valdivia 2008). Transcriptomic (Belland *et al*. 2003) and proteomic (Saka *et al*. 2011) studies revealed that CT694 and CT695 are expressed late in the developmental cycle and are strictly present in EBs. Both CT694 and CT695 are secreted in a T3SS-dependent manner and associate with host membranes, earning their designation translocated membrane-associated effector A (TmeA) and B (TmeB), respectively (Sisko *et al*. 2006; Hower, Wolf and Fields 2009; Pais *et al*. 2013; Mueller and Fields 2015; Keb, Hayman and Fields 2018).

Recent advances in chlamydial genetics confirmed that TmeA plays an important role in host cell invasion, whereas TmeB appears to be dispensable for pathogen uptake (McKeun *et al*. 2017; Keb, Hayman and Fields 2018). Using a yeast two-hybrid (Y2H) screen, TmeA was shown to bind AHNAK, a large scaffolding protein involved in cytoskeletal organization and cell signaling (Hower, Wolf and Fields 2009). While TmeA is necessary for host cell invasion, AHNAK is dispensable. Furthermore, AHNAK is still recruited to the invasion site in the absence of TmeA (McKeun *et al*. 2017). Thus, the role TmeA plays in promoting host cell invasion is independent of its interaction with AHNAK (Fig. 3).

New research indicates that TmeA possesses a GTPase binding domain (GBD) ligand motif that binds to the NPF N-WASP (Faris *et al*. 2020). Interactions between N-WASP and TmeA promote recruitment of the ARP2/3 complex to the invasion site, presumably driving actin branching events necessary for filopodia capture and internalization of EBs (Faris *et al*. 2020) (Fig. 3). Importantly, complementation of the TmeA mutant with a mutant GBD ligand motif did not restore invasion, confirming that it is TmeA's interaction with N-WASP that promotes host cell invasion. It is compelling to speculate that TmeA may serve two distinct functions. First, during invasion, TmeA activates N-WASP, leading to ARP2/3-dependent cytoskeletal remodeling necessary for filopodia capture of *C. trachomatis* EBs. Second, following invasion, TmeA could interact with AHNAK to undo the actin-bundling effects induced during the invasion process (Caven and Carabeo 2020).

While TmeA was shown to be necessary for N-WASP recruitment to the invading EB, the ARP2/3 complex was still recruited in the absence of TmeA and N-WASP, albeit to a significantly lower degree (Faris *et al*. 2020). This suggests that *C. trachomatis* employs multiple methods to recruit the ARP2/3 complex to the EB invasion site. Indeed, a TarP mutant was similarly impaired in ARP2/3 recruitment, whereas recruitment to a TmeA/TarP double mutant was completely abolished (Faris *et al*. 2020). TarP was previously shown to bind Rac GEFs and it was hypothesized that this leads to activation of WAVE2 and the ARP2/3 complex (Carabeo *et al*. 2007; Lane *et al*. 2008). In aggregate, these studies suggest that *C. trachomatis* employs two distinct effector proteins, TarP and TmeA, that target distinct NPFs that ultimately converge on activation of the ARP2/3 complex to drive actin branching events required for host cell invasion. Curiously, the TmeA/TarP double mutant was significantly impaired in host cell invasion, yet a small percentage of EBs still gained access to host cells. This highlights a crucial role for TarP and TmeA in invasion via manipulation of the ARP2/3 complex, while indicating that additional host factors and bacterial effectors may be involved in host cell invasion. Given the recent advances in chlamydial genetics, it will be interesting to determine whether other pre-packaged effector proteins play a role in host cell invasion. Furthermore, it will be of great interest to determine whether these different effectors and invasion pathways contribute to *C. trachomatis* cellular/tissue tropism.

## MOVEMENT TO THE MICROTUBULE-ORGANIZING CENTER AND ASSOCIATION WITH CENTROSOMES

At ∼2 h post-infection, the nascent inclusion is transported in a dynein-dependent manner to the microtubule-organizing center (MTOC) (Clausen *et al*. 1997; Grieshaber, Grieshaber and Hackstadt 2003). This process requires an intact microtubule network and chlamydial protein synthesis (Grieshaber, Grieshaber and Hackstadt 2003), suggesting bacterial effector proteins could tether the inclusion to dynein or centrosomes. Several *C. trachomatis* Inc proteins, including CT101, CT222, CT223, CT224, CT228, IncB, IncC, CT228 and CT850, are concentrated in areas on the inclusion membrane referred to as microdomains (Mital *et al*. 2010; Weber *et al*. 2015). These areas are enriched in cholesterol, active Src-family kinases, and are focal points for microtubules and association with centrosomes (Mital *et al*. 2010). Thus, these Inc proteins could play a role in forming stable interactions between the inclusion and microtubules or centrosomes.

**Figure 4.**
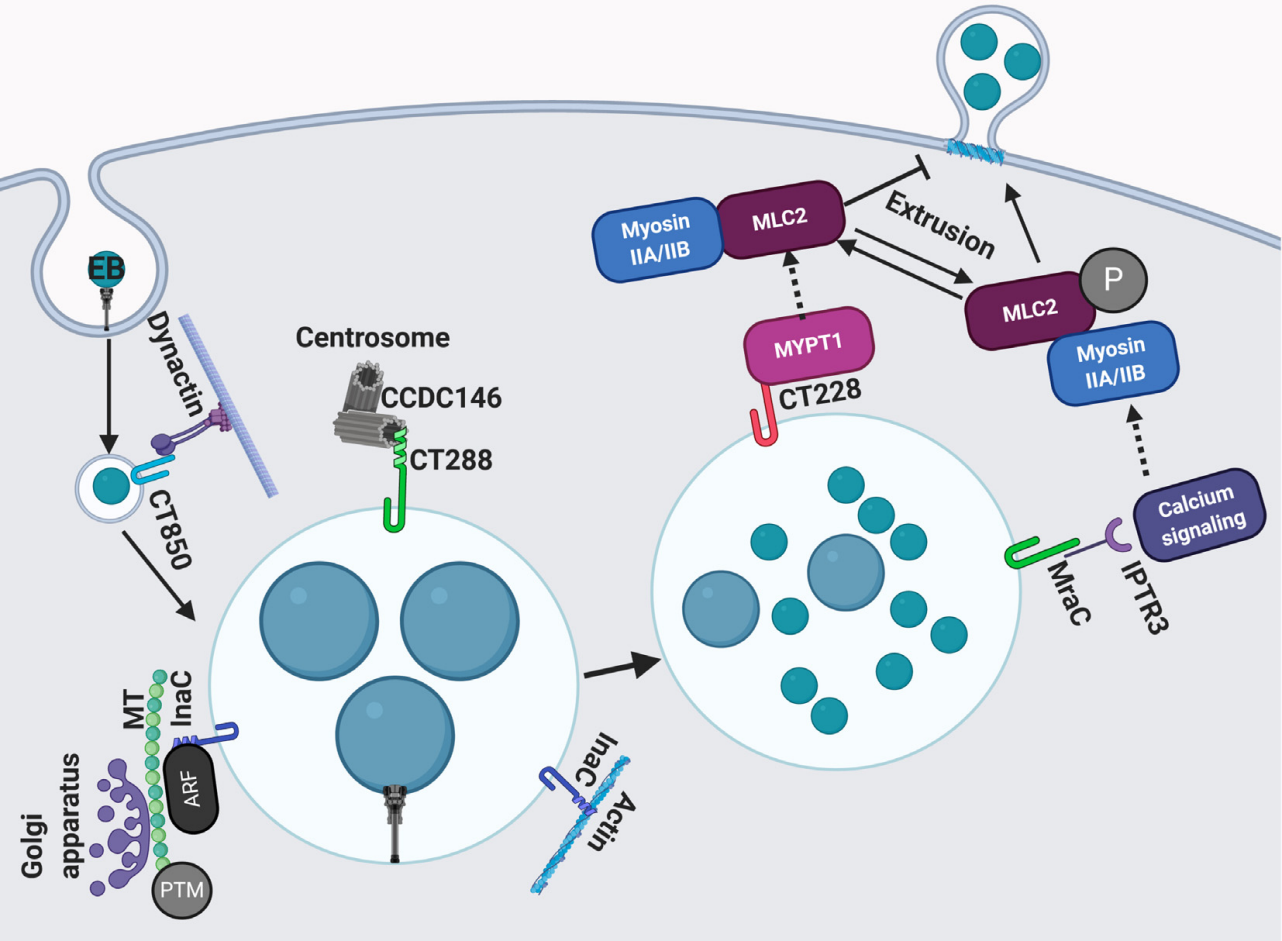
Interactions with centrosomes and subversion of the cytoskeleton by Inc proteins. The Inc protein CT850 interacts with dynein light-chain DYNLT1 to facilitate inclusion positioning at the MTOC. CT288 binds to the centrosomal protein CCDC146. InaC is a multifunctional Inc that interacts with ARF GTPases to control Golgi positioning at the inclusion. InaC is also important for F-actin recruitment to the inclusion. CT228 and MraC inhibit or promote extrusion, respectively, through regulation of MLC2 phosphorylation state.

When ectopically expressed in HeLa cells, CT850 aggregates were found to associate with centrosomes (Mital *et al*. 2010), suggesting this Inc protein could play a role in inclusion positioning at the MTOC. Using a Y2H screen, CT850 was shown to interact with dynein light-chain DYNLT1 via a conserved (R/K-R/K-X-X-R/K) DYNLT1 binding domain (Mital *et al*. 2015). DYNLT1 localizes to the inclusion membrane and disruption of DYNLT1 expression interferes with inclusion positioning at the MTOC. The CT850-DYNLT1 interaction suggests that *C. trachomatis* may subvert the dynein motor to move the inclusion along microtubules in the absence of an intact dynactin complex (Fig. 4). While lack of CT850 does not impair growth (Weber *et al*. 2017), whether insertional inactivation of CT850 impairs inclusion trafficking to the MTOC remains unknown.

As inclusion microdomains localize near the host centrosome (Mital *et al*. 2010), it is not surprising that multiple microdomain-localized Incs interact with the centrosome. A Y2H screen revealed the microdomain-localized Inc CT288 binds the centrosome protein CCDC146 (Almeida *et al*. 2018). CCDC146 localized proximal to the inclusion; however, this was only partially dependent on CT288, as CCDC146 was still recruited to a CT288 mutant (Almeida *et al*. 2018) (Fig. 4). Many questions remain unanswered regarding whether CT288 and its interaction with CCDC146 has any impact on inclusion positioning at the centrosome. While the relevance of CCDC146 to *Chlamydia* infection remains unknown, one study noted a slight *in vitro* and *in vivo* growth defect when CT288 is absent (Weber *et al*. 2017), suggesting this Inc may play a role in *Chlamydia* infection.

## REGULATION OF FUSION AND MANIPULATION OF HOST VESICLE TRAFFICKING

From the confines of the inclusion, chlamydiae modulate specific aspects of host intracellular trafficking and fusogenicity with the inclusion in order to acquire membrane for the growing vacuole and essential nutrients. Given their positioning at the host-pathogen interface, it is not surprising that Inc proteins have risen to prominence as key regulators of host vesicular trafficking and fusion. Additionally, a few secreted effector proteins have been implicated in manipulating vesicular trafficking pathways.

In 1994, the observation was made that protein was released from chlamydiae and localized at the inclusion membrane (Rockey and Rosquist 1994). Screening of a *C. psittaci* expression library with convalescent sera resulted in the identification of this protein as inclusion membrane protein A (IncA) (Rockey, Heinzen and Hackstadt 1995), which was subsequently shown to also be present in *C. trachomatis* (Bannantine *et al*. 1998). Microinjection of antibodies against IncA into cells infected with *C. trachomatis* significantly altered inclusion morphology, resulting in multiple inclusions per cell (Hackstadt *et al*. 1999). This suggested that IncA could be involved in homotypic fusion of inclusions. Further support for this notion came via screening clinical isolates, of which ∼1.5% exhibited multiple inclusions per cell. Immunofluorescent microscopy and western blotting of these isolates revealed they lacked IncA (Suchland *et al*. 2000). A role for IncA in mediating homotypic fusion of inclusions was later confirmed through generation of an IncA mutant using the TargeTron approach (Johnson and Fisher 2013).

**Figure 5.**
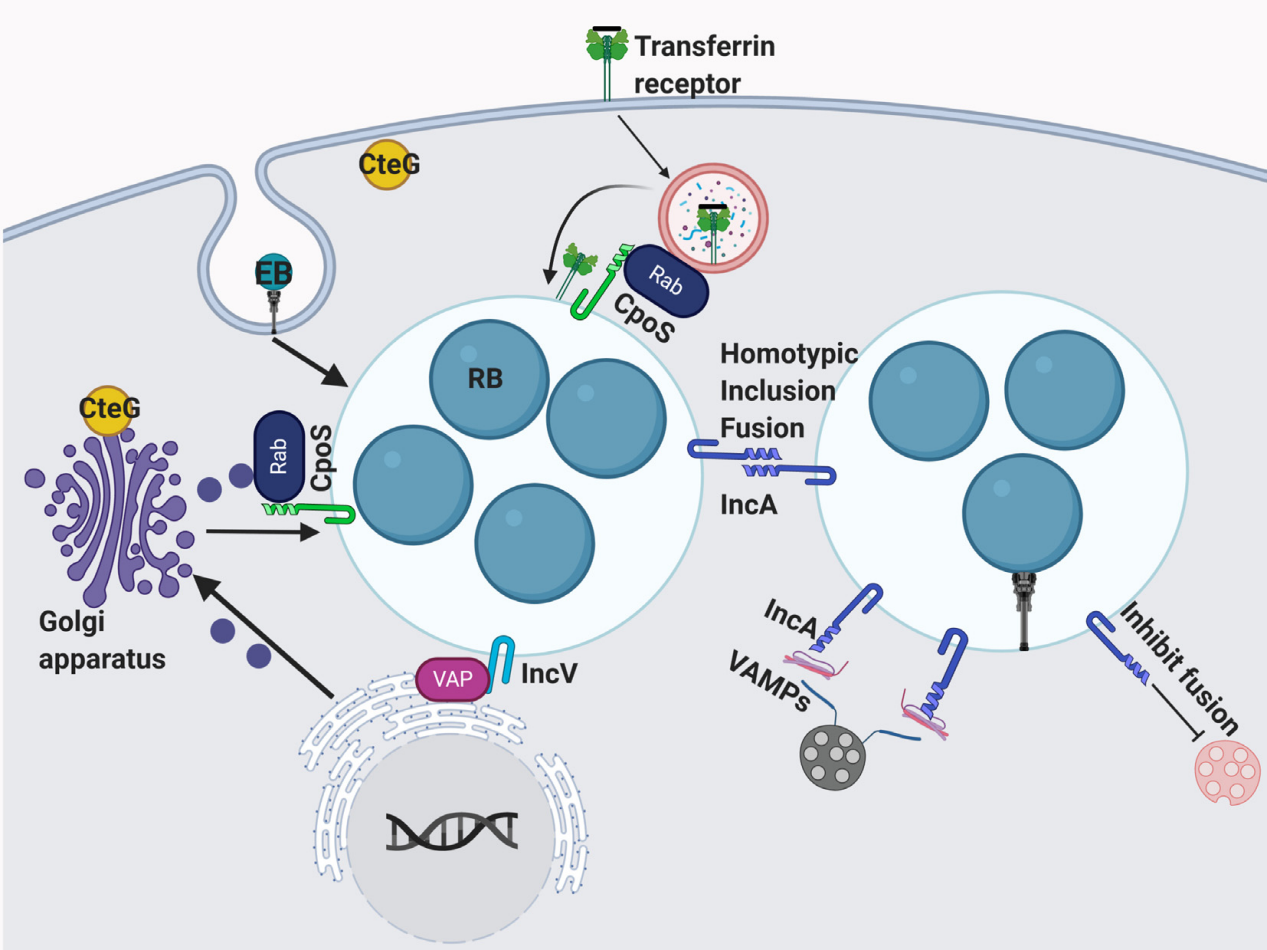
Manipulation of host vesicular trafficking by Inc and cT3SS effector proteins. CpoS interacts with Rab GTPases to recruit the transferrin receptor to the inclusion. IncV, through interactions with VAPs, functions to tether the inclusion to the ER. CteG localizes to the Golgi apparatus and plasma membrane and may be involved in regulating trafficking. IncA is involved with homotypic inclusion fusion and interacts with several VAMPs.

Observations that regions of IncA are exposed to the host cytoplasmic space suggested that Inc proteins could mediate crucial interactions between the host and the bacteria confined within the inclusion (Rockey *et al*. 1997). Modeling of IncA revealed it possesses soluble *N*-ethylmaleimide-sensitive factor attachment protein receptor (SNARE)-like domain (SLD) composed of heptad repeat sequences consisting mostly of hydrophobic residues with a conserved glutamine (Q-SNARE) or arginine (R-SNARE) residue defining the zero layer (Delevoye *et al*. 2004, 2008). In eukaryotes, SNARE proteins in opposing lipid bilayers associate to form a complex that can promote or inhibit fusion between compartments (Cai, Reinisch and Ferro-Novick 2007). As key regulators of membrane fusion, it is not surprising that intracellular pathogens have effector proteins that possess SNARE-like domains (Arasaki, Toomre and Roy 2012; Singh *et al*. 2018). Intriguingly, IncA possesses two SLDs: SLD1 and SLD2. Biochemical analysis revealed that a functional core composed of SLD1 and part of SLD2 is required to promote homotypic inclusion fusion, whereas either SLD is able to block membrane fusion (Ronzone and Paumet 2013; Ronzone *et al*. 2014) (Fig. 5). The necessity of the IncA core in mediating homotypic inclusion fusion was confirmed using an IncA mutant complemented with IncA lacking a functional core domain (Weber *et al*. 2016). Structural analysis of IncA indicates that it folds differently than the canonical four-helix bundle associated with SNAREs and instead resembles the THATCH domain of the Huntingtin-interacting protein 12, a component of clathrin-coated pits. Thus, IncA may serve to link endocytic components with the actin cytoskeleton (Cingolani *et al*. 2019).

It is well established that the chlamydial inclusion avoids fusion with endocytic and lysosomal compartments (Heinzen *et al*. 1996; Scidmore, Fischer and Hackstadt 2003). Early endocytic/lysosomal avoidance occurs before IncA expression, which is not expressed until ∼10–12 h post-infection. Thus, IncA may not be involved in initial avoidance, but could inhibit fusion with these compartments later in the infection cycle. With the advances in chlamydial genetics and methods to complement mutants with domain mutants, it will now be feasible to determine whether SLD1 or SLD2 is important for avoiding fusion with endocytic compartments. IncA's important role in infection is distinct, as infection with non-fusogenic isolates results in milder disease (Geisler *et al*. 2001; Pannekoek *et al*. 2005).

Fusion between vesicles requires the formation of a four-helix bundle formed through associations of vesicular SNAREs (v-SNAREs) and target SNAREs (t-SNAREs), which provides the necessary energy to drive fusion of the membrane bilayers (Cai, Reinisch and Ferro-Novick 2007). A number of eukaryotic SNAREs have been shown to localize to the inclusion membrane and disruption of these SNAREs impairs sphingomyelin and lipid droplet recruitment to the inclusion, coinciding with impaired bacterial replication (Delevoye *et al*. 2008; Moore *et al*. 2011; Kabeiseman *et al*. 2013; Monteiro-Brás, Wesolowski and Paumet 2020). IncA was shown to bind VAMP3, VAMP7 and VAMP8 (Delevoye *et al*. 2008), and furthermore was shown to inhibit fusion with liposomes harboring VAMP8, Syntaxin 7, Syntaxin 8 and Vti1b (Paumet *et al*. 2009) (Fig. 5). Other SNARE proteins, including SNAP23, Syntaxin 4 and Syntaxin 6, are recruited to the inclusion (Moore *et al*. 2011; Kabeiseman *et al*. 2013; Monteiro-Brás, Wesolowski and Paumet 2020). However, whether this is through interactions with IncA or another Inc protein remains unknown. With the isolation of an IncA mutant (Johnson and Fisher 2013; Weber *et al*. 2016), it will now be possible to test the requirement of IncA for recruitment of eukaryotic SNAREs, as well as the functional consequences of IncA loss. While IncA appears to play an important role in inhibiting fusions with vesicles containing select SNAREs, it is likely that *C. trachomatis* has compensatory mechanisms for avoiding fusion with these compartments, as loss of IncA does not negatively impact growth (Johnson and Fisher 2013; Weber *et al*. 2016).

Movement of vesicular cargo from one region of the cell to another is a tightly regulated process that is controlled by SNAREs and small guanosine triphosphate (GTP) binding proteins including Rab GTPases and ADP-ribosylating factors (ARFs) (Weber and Faris 2018). Rab GTPases associated with trafficking from the Golgi apparatus (Rab1, 6 and 10) and early endosomes (Rab4 and 11) are recruited to the inclusion membrane (Rzomp *et al*. 2003). Using a Y2H screen and pulldowns, Rzomp *et al*. demonstrated that the Inc protein CT229 binds to Rab4 (Rzomp, Moorhead and Scidmore 2006). Subsequent studies using *C. trachomatis* overexpressing Flag-tagged CT229 (Sixt *et al*. 2017; Faris *et al*. 2019) or cells transfected with CT229 (Mirrashidi *et al*. 2015) revealed it binds and recruits a plethora of Rab GTPases, including Rab1, 2, 4, 6, 8, 10, 14, 18, 33, 34 and 35 to the inclusion membrane or vicinity of the inclusion. Furthermore, CT229, through binding to Rab GTPases, was shown to recruit the transferrin receptor and cation-independent mannose-6-phosphate receptor to the periphery of the inclusion (Faris *et al*. 2019) (Fig. 5). In aggregate, these studies implicate CT229 as an important regulator of host vesicular trafficking and suggest it might play a role in nutrient and/or membrane acquisition for the growing inclusion.

The isolation of a CT229 mutant using chemical mutagenesis and TargeTron revealed that CT229 plays an important role in chlamydial pathogenesis (Kokes *et al*. 2015; Sixt *et al*. 2017; Weber *et al*. 2017). The absence of this Inc protein results in decreased bacterial replication, smaller inclusions and faster clearance *in vivo* (Sixt *et al*. 2017; Weber *et al*. 2017). Strikingly, the absence of CT229 resulted in premature host cell death, resulting in its designation as *Chlamydia*promoter of survival (CpoS). Descriptive studies aimed at characterizing the mode of host cell death elicited in the absence of CpoS revealed host cells displayed hallmarks of apoptosis, characterized by membrane blebbing and activation of effector caspases (Sixt *et al*. 2017; Weber *et al*. 2017). Surprisingly, some of the cells also displayed characteristics of necrosis in which plasma membrane rupture was not preceded by apoptotic features (Sixt *et al*. 2017). Infection of host cells with a CpoS mutant elicited a STING-dependent cytokine response associated with TNF-α and type-1 interferon (IFN) production, in addition to up-regulation of IFN-stimulated genes (Sixt *et al*. 2017). Knockdown of STING partially protected the cells from premature host cell death induced in response to infection with the CpoS mutant (Sixt *et al*. 2017; Weber *et al*. 2017). Thus, while this pathway is involved in recognizing CpoS-deficient chlamydiae, it is not the sole factor that elicits host cell death in response to the mutant bacteria. While the absence of CpoS triggers premature host cell death, the underlying reason for this remains unclear. Recent studies indicate CpoS is necessary for recruiting Rab GTPases to the inclusion (Faris *et al*. 2019). STING activation requires translocation via post-ER vesicles (Sixt, Valdivia and Kroemer 2017). Given that CpoS interacts with Rab GTPases, it is possible that CpoS-mediated manipulation of host vesicular trafficking pathways counters STING activation during chlamydial infection. The absence of CpoS was shown to result in premature inclusion lysis, which would result in the release of the bacteria and their components into the cytosol where they can be sensed by host surveillance pathways. *Chlamydiatrachomatis* synthesizes cyclic di-AMP, which can be sensed by STING, resulting in production of type I IFNs (Barker *et al*. 2013). We speculate that the manipulation of host vesicular trafficking via CpoS-Rab GTPases interactions could supply crucial membrane and lipids for incorporation into the expanding inclusion membrane, the absence of which could lead to premature inclusion lysis and release of cyclic di-AMP into the host cytosol where it is sensed by STING. Other Inc mutants exhibit similar phenotypes characterized by destabilization of the inclusion (Weber *et al*. 2017; Giebel *et al*. 2019), and laser ablation of the inclusion results in induction of premature host cell death (Kerr *et al*. 2017). Thus, while it is likely that some chlamydial effectors play a key role in counteracting host cytokine production, the fact that premature host cell death and STING activation could be a general consequence of chlamydiae in the cytosol is also possible.

CT105 is a cT3SS effector protein that localizes to the Golgi apparatus early in infection (16–30hr) and the plasma membrane later in infection (30–40 hr), earning its designation *C. trachomatis*effector associated with the Golgi (CteG) (Pais *et al*. 2019) (Fig. 5). While a CteG mutant was not impaired in intracellular replication, smaller inclusions were noted (Pais *et al*. 2019). Currently, the host targets of CteG remain unknown; however, ectopic expression of CteG in yeast induces a vacuolar protein sorting defect, indicating CteG could modulate host vesicular trafficking (Pais *et al*. 2019). Intriguingly, CteG from non-LGV chlamydial isolates (serovars A–K) lack 74 nucleotides upstream from the putative −10 region resulting in lack of expression. Hence, CteG is only expressed by LGV isolates (L1–L3). Future studies are needed to determine whether CteG carries out distinct functions early in infection when associated with the Golgi, versus late in infection when associated with the plasma membrane.

## FORMATION OF MEMBRANE CONTACT SITES

*Chlamydia*spp. must hijack host lipids, including cholesterol, sphingomyelin, phosphatidylcholine and phosphatidylinositol for incorporation into the bacterial membrane (Hackstadt, Scidmore and Rockey 1995; Hackstadt *et al*. 1996; Wylie, Hatch and Mcclarty 1997; Hatch and Mcclarty 1998; Carabeo, Mead and Hackstadt 2003). While *C. trachomatis* can acquire lipids from Golgi mini-stacks or multivesicular bodies, it is apparent that non-vesicular transport pathways can also be manipulated through the formation of membrane contact sites (MCS) between the membrane of the parasitophorous vacuole and the host organelle (Derré 2017).

Vesicle-associated membrane protein associated-protein (VAP) A and B (VAPA and VAPB) participate in the formation of MCS between the ER and other organelles (Derré 2017). VAPs bind to proteins that possess a 7 amino acid FFAT motif. As the chlamydial inclusion forms MCS with the ER, it is not surprising that VAPs localize to the inclusion membrane (Derré, Swiss and Agaisse 2011). A large-scale proteomic screen detected an interaction between VAPA/B and the inclusion membrane protein CT005 (Mirrashidi *et al*. 2015) (Fig. 5). Due to its interaction with VAPs, CT005 is now designated as IncV for Inc interaction with VAP (Stanhope *et al*. 2017). The C-terminus of IncV possesses two FFAT motifs, which were shown to be necessary for binding to VAPs (Stanhope *et al*. 2017). While an IncV null mutant is not impaired in intracellular replication, it does exhibit reduced VAP recruitment, and siRNA knockdown of VAPA and VAPB reduces inclusion size and infectious progeny production (Derré, Swiss and Agaisse 2011; Stanhope *et al*. 2017; Weber *et al*. 2017). Given that IncV intercalates into the inclusion membrane where it can interact with VAPs at ER-inclusion MCS and overexpression of IncV enhances VAP recruitment suggests that IncV functions as a tether (Stanhope *et al*. 2017) (Fig. 5). While IncV clearly plays a role in ER-inclusion tethering, ER-inclusion MCS and VAP recruitment are not abolished in the absence of IncV, suggesting other chlamydial factors could also function as tethers.

## REORGANIZATION OF THE CYTOSKELETON

In order to maintain the stability and structure of the chlamydial inclusion, *C. trachomatis* co-opts the function of all four cytoskeletal elements: microtubules (MT), actin, intermediate filaments (IF) and septins (Kumar and Valdivia 2008; Al-Zeer *et al*. 2014; Volceanov *et al*. 2014). IFs have been implicated in providing stability to the expanding inclusion, whereas MTs promote movement of the inclusion to the MTOC (Grieshaber, Grieshaber and Hackstadt 2003; Kumar and Valdivia 2008). In contrast, actin polymerization has been implicated in invasion and release of chlamydial EBs.

Screening of a *C. trachomatis* chemical mutant library using a microscopy-based approach revealed that a CT813 mutant is deficient in recruiting F-actin to the inclusion (Kokes *et al*. 2015). As the loss of CT813 results in a loss of F-actin recruitment to the inclusion, it was renamed InaC for inclusion membrane protein for actin assembly (Kokes *et al*. 2015). By immunoprecipitating GFP-tagged InaC from transfected cells, it was determined that InaC interacts with members from two different protein families: ADP-ribosylation factors (ARF1, 4 and 5) and 14-3-3 proteins (Kokes *et al*. 2015; Wesolowski *et al*. 2017). Intriguingly, another study demonstrated that only ARF1 and 4 binds to InaC when it is overexpressed in *Chlamydia*. Furthermore, only ARF1 and 4 were recruited to the inclusion in an InaC-dependent manner (Wesolowski *et al*. 2017). These experimental differences could be due to differences in tags (GFP vs. Flag) or other experimental factors. The absence of InaC or knockdown of ARF1 or 4 impaired Golgi distribution around the inclusion in a manner that required intact F-actin filaments (Kokes *et al*. 2015; Wesolowski *et al*. 2017) (Fig. 4). While the total amount and structure of MT cages was not effected by loss of InaC, the amount of post-translationally modified MTs was strikingly decreased (Wesolowski *et al*. 2017). This observation corroborates previous studies that demonstrated that the amount of detyrosinated and acetylated alpha-tubulin increases during *Chlamydia* infection (Al-Zeer *et al*. 2014). While InaC obviously plays an important role in controlling Golgi-positioning via binding to ARFs, the mechanistic underpinnings of this interaction remain unknown. Interestingly, InaC does not function as a guanine-nucleotide exchange factor, implying it must be able to activate ARFs via a unique mechanism (Wesolowski *et al*. 2017). ARFs clearly play a role in formation of the MT nest that encases the inclusion; however, other host factors have been implicated in formation of actin scaffolds (Kumar and Valdivia 2008; Paumet and Wesolowski 2017). Future work is needed to understand how InaC coordinates recruitment and formation of both F-actin and MT scaffolds at the inclusion.

## MANIPULATION OF THE HOST CELL DEATH AND THE IMMUNE RESPONSE

Chaperones often associate with T3SS effector proteins to promote effector translocation, a feature that has been exploited to identify candidate effector proteins (Pais *et al*. 2013; Chen *et al*. 2014). *Chlamydia trachomatis* possesses at least six putative T3SS chaperones (Chen *et al*. 2014). The secreted effector proteins TarP, TmeA and TmeB have been shown to share the chaperone Slc1, suggesting Slc1 may mediate translocation of additional effector proteins (Pais *et al*. 2013). By immunoprecipitating Slc1 and McsC from EB lysates, Chen *et al*. identified several novel candidate effector proteins, including CT875 (Chen *et al*. 2014).

Similar to TarP, CT875 is tyrosine phosphorylated by host Src family kinases following delivery into the eukaryotic cell, earning its designation translocated early phosphoprotein (TepP) (Chen *et al*. 2014). Tyrosine phosphorylation of TepP provides a docking site for SH2 and SH3 proteins, such as the signaling adaptor proteins Crk (Crk-I and Crk-II), Crk-like proto-oncogene adaptor protein (CrkL), glycogen synthase kinase 3β (GSK3B) and class I phosphoinositide 3-kinases (PI3K) (Chen *et al*. 2014; Carpenter *et al*. 2017). Both CrkL and PI3K participate in immune signaling via interaction with STAT5, resulting in a type I interferon response. While TepP is dispensable for host cell invasion, it appears to play a key role in modulating host gene expression early in infection. Global transcriptomic profiling of endocervical epithelial cells infected with a TepP mutant identified 33 genes, many with immunity-related functions, that are differentially expressed compared with a wild-type infection. Notably, this included reduced expression of IFN-induced peptides with the tetratricopeptide repeat (*IFIT1* and *IFIT2*) genes, which play an integral role in the anti-viral response. Induction of *IFIT* expression following *Chlamydia* infection was later linked to PI3K activity in the vicinity of the nascent inclusion (Carpenter *et al*. 2017). TepP was also shown to dampen the expression of chemokines (IL-6 and CXCL3), which promotes recruitment of neutrophils (Chen *et al*. 2014). Using an endometrial organoid model infected with a TepP mutant, Dolat and Valdivia demonstrated that TepP serves to dampen the immune response to *C. trachomatis* infection by limiting the influx of neutrophils (Dolat and Valdivia 2020).

## EXITING THE HOST CELL

At the conclusion of the developmental cycle, bacteria are released by host cell lysis or extrusion (Hybiske and Stephens 2007). Given that the inclusion is encased in a complex cytoskeletal meshwork composed of MTs, IFs, actin and septins, it is not surprising that release requires manipulation of the cytoskeleton to extradite the inclusion from its cage. The extrusion route is dependent upon the myosin light chain 2 (MLC2) phosphorylation state, which is regulated by myosin kinase (MLCK) and myosin phosphatase (MYPT1) (Bugalhão and Mota 2019). Lysis is favored when MLC2 is dephosphorylated, whereas extrusion is favored when MLC2 is phosphorylated (Lutter *et al*. 2013).

Using a Y2H screen, Lutter *et al*. demonstrated that the Inc protein CT228 binds to MYPT1 (Lutter *et al*. 2013). Phosphorylated MYPT1 was recruited to the periphery of the inclusion and both CT228 and MYPT1 were enriched in inclusion microdomains (Mital *et al*. 2010; Lutter *et al*. 2013). Phosphorylation of MYPT1 at T696 and T853 induces MYPT1 folding such that it can no longer interact with MLC2. As this would prevent dephosphorylation, MLC2 is still able to interact with myosin IIA and IIB, promoting extrusion (Lutter *et al*. 2013) (Fig. 4). Phosphorylated MLC2 and MLCK, the kinase that regulates the phosphorylation state of MLC2, was also observed at microdomains. Microdomains are areas on the inclusion that serve as focal points for the recruitment of Src-family tyrosine kinases, which are known to phosphorylate MLCK. Thus, microdomains could serve as signaling platforms that controls bacterial egress from the cell. A CT228 mutant was unable to recruit MYPT1, correlating with increased extrusion (Shaw *et al*. 2018). Surprisingly, CT228 disruption did not affect MLC2 recruitment, implying that *C. trachomatis* may possess other factors that mediate MLC2 recruitment and host cell egress. Intriguingly, clearance of a CT228 mutant was delayed during murine intravaginal infection, suggesting host cell escape via extrusion impacts the duration of *in vivo* infection (Shaw *et al*. 2018).

Another Inc, MrcA (CT101), recruits the Ca^2+^ channel inositol-1,4,5-trisphosphate receptor type 3 (ITPR3) to microdomains, where it localizes with active Src-family tyrosine kinases and STIM1, a Ca^2+^ sensor situated on the ER (Nguyen, Lutter and Hackstadt 2018). An MrcA mutant was unable to recruit ITPR3 and exhibited reduced extrusion (Fig. 4). Extrusion was also inhibited by siRNA knockdown of ITPR3 or STIM1, as well as by the calcium chelator BAPTA-AM. Decreased extrusion correlated with decreased MLC2 phosphorylation and depletion of myosin motor activity. Ultimately, this highlights the critical role of Ca^2+^ signaling pathways in the activation of chlamydial extrusion, as well as confirming that microdomains serve as hubs for cytoskeletal interactions.

## CONCLUSIONS

*Chlamydia trachomatis* is presumed to deliver over 100 proteins through its T3SS that interfere with normal host cell processes to promote invasion, intracellular replication, inclusion formation and dissemination. The development of genetic tools to express epitope-tagged proteins and to make site-specific or random mutants in *Chlamydia* has substantially enhanced our understanding of how this important pathogen forms and maintains its niche within host cells. While the ability to make mutants has allowed us to link effector function to bacterial pathogenesis, the ability to manipulate chlamydiae remains challenging. For several important Incs, including IncD and IncE, the molecular function has been addressed using biochemical and molecular techniques (Derré, Swiss and Agaisse 2011; Mirrashidi *et al*. 2015), while the generation of a mutant in these effector proteins has been a major hurdle. These important effectors, along with many others, may be essential to chlamydial development. The ability to make conditional mutants using CRISPRi (Ouellette 2018) may be very beneficial to address these issues. Regardless, a detailed understanding of how this important pathogen subverts the host to establish its privileged niche is important for the development of improved therapeutics to combat infection.

## FUNDING

This work is supported by grants from the National Institutes of Health (1R01AI150812 and 1R01AI155434-01) to MMW.
